# Nursing‐Led Support Across the Standard Care–Clinical Trial–Standard Care Pathway in Inflammatory Bowel Disease: A Review With Realist‐Informed Interpretation

**DOI:** 10.1002/jgh3.70458

**Published:** 2026-08-19

**Authors:** Daniele Napolitano, Mattia Bozzetti, Vincenzina Mora, Francesco Pastore, Rosario Caruso, Monica Guberti, Valeria Amatucci, Loris Riccardo Lopetuso, Lucrezia Laterza, Franco Scaldaferri, Antonio Gasbarrini

**Affiliations:** ^1^ Scientific Direction, Fondazione Policlinico Universitario “A. Gemelli” IRCCS Rome Italy; ^2^ Department of Translational Medicine and Surgery Università Cattolica del Sacro Cuore Rome Italy; ^3^ Direction of Health Professions, ASST Cremona Cremona Italy; ^4^ Department of Precision and Regenerative Medicine University of Bari Aldo Moro Bari Italy; ^5^ Department of Biomedical Sciences for Health University of Milan Milan Italy; ^6^ Health Professions Research and Evidence Transfer Unit IRCCS MultiMedica Milan Italy; ^7^ Directorate of Nursing and Allied Health Professions, IRCCS Istituto Ortopedico Rizzoli Bologna Italy; ^8^ CEMAD Fondazione Policlinico Universitario “A. Gemelli” IRCCS Rome Italy; ^9^ Department of Life Sciences, Health and Health Professions Link Campus University Rome Italy

**Keywords:** clinical trials as topic, inflammatory bowel diseases, nursing care, patient compliance, telemedicine

## Abstract

**Aims:**

To examine how nursing‐led support may influence engagement, continuity, adherence, retention, and protocol enactment across the standard care–clinical trial–standard care pathway in individuals with inflammatory bowel disease (IBD).

**Methods and Results:**

This focused narrative review used selected realist‐informed interpretive principles as a conceptual lens to synthesize heterogeneous literature related to IBD clinical trials, specialist and clinical research nursing, placebo and nocebo processes, health literacy, teach‐back, telemonitoring, continuity of care, transitions, implementation, and intervention fidelity. Rather than conducting a formal realist review, realist concepts were used to inform the interpretation of how contextual factors, participant reasoning, and implementation conditions may influence nursing‐led support across the clinical trial pathway. Literature was identified through iterative searches in PubMed/MEDLINE, Scopus, and Google Scholar, complemented by backward and forward citation tracking. Five interrelated supportive functions emerged across the reviewed literature: expectation‐shaping during screening and enrolment; literacy‐sensitive education and teach‐back; structured hybrid contact and continuity support; transition‐sensitive assistance during trial entry, amendment, and exit phases; and governance strategies promoting role clarity, safety, and equity. Nursing‐led support appeared most relevant when it helped participants interpret uncertainty, understand protocol expectations, maintain continuity, and navigate vulnerable transitions. These functions may contribute to engagement, adherence, retention, and timely symptom reporting by strengthening trust, self‐efficacy, relational continuity, and adherence to practical protocols.

**Conclusion:**

Nursing‐led support may represent a potential mechanism‐bearing component of IBD clinical trial participation rather than a purely administrative activity. Transition‐sensitive, literacy‐aware, and continuity‐oriented nursing strategies may support safer, more equitable, and more sustainable engagement across increasingly complex clinical trial pathways.

## 1 Introduction

Clinical trials have become increasingly complex and communication‐intensive, and inflammatory bowel disease (IBD) studies exemplify this trend. Recruitment is often challenging, screen‐failure rates are high, and study protocols commonly include repeated endoscopy, demanding visit schedules, digital monitoring, washout periods, and uncertainty regarding placebo exposure. These factors affect not only recruitment and retention but also participants' experience of uncertainty, symptom interpretation, and willingness to remain engaged throughout trial participation [1, 2, 3, 4].

IBD is a relapsing–remitting condition in which symptom perception, treatment beliefs, health literacy, and continuity of care influence how patients interpret therapeutic experiences. Placebo and nocebo responses further highlight the importance of contextual factors during trial participation. Because nurses often provide the most frequent participant contact, their communication may represent an important contextual component influencing expectations, symptom interpretation, and engagement throughout the clinical trial pathway [5, 6, 7, 8, 9].

Clinical Research Nurses (CRNs) and specialist IBD nurses are particularly well positioned to provide this support [1, 10, 11]. Their activities include therapeutic education, symptom triage, continuity of care, digital onboarding, expectation alignment, transition support, and trial preparation, including feasibility assessment, protocol operationalisation, and the development of participant‐facing procedures [10, 12]. Collectively, these functions may facilitate recruitment, protocol adherence, participant engagement, retention, and continuity across the interface between clinical care and research [10, 13, 14, 15, 16].

Although specialist IBD nurses and CRNs are distinct professional roles in many healthcare systems, they share several patient‐centered supportive functions relevant to this review. Specialist IBD nurses primarily provide long‐term disease management, therapeutic education, symptom monitoring, and continuity of care within routine clinical practice, whereas CRNs focus on protocol implementation, participant safety, regulatory compliance, trial coordination, and research delivery. Depending on the healthcare system, these responsibilities may be undertaken by separate professionals or shared across roles. Accordingly, this review focuses on nursing‐led supportive functions across the clinical trial pathway rather than attributing all activities to a single professional role [2, 17, 18, 19, 20, 21].

This review examines how nursing‐led support may influence engagement, adherence, retention, symptom sense‐making, and continuity across the standard care–clinical trial–standard care pathway in IBD. Given the heterogeneous nature of the available evidence, the review was designed as a focused narrative synthesis using selected realist‐informed concepts solely as an interpretive lens. The objective was to synthesize heterogeneous evidence and develop a preliminary theory‐informed framework rather than to conduct a formal realist synthesis or generate Context–Mechanism–Outcome (CMO) configurations.

## 2 Methods

### 2.1 Review Design and Realist Rationale

This study was designed as a focused narrative review using selected realist‐informed interpretive principles. The review did not follow formal realist review methodology or the RAMESES publication standards. Instead, realist concepts were used as a methodological sensitizing framework to interpret heterogeneous evidence by considering how contextual conditions, participant reasoning, and implementation factors may influence the potential contribution of nursing‐led support across the clinical trial pathway. Consequently, no formal CMO configurations were developed or tested. This focused narrative review was developed with reference to the Scale for the Assessment of Narrative Review Articles (SANRA) (Supporting Information).

### 2.2 Review Question

The review asked how nursing‐led communication, education, continuity of care, telemonitoring, and transition support may influence engagement, adherence, retention, participant experience, and protocol enactment across the standard care–trial–standard care pathway in IBD.

### 2.3 Evidence Corpus, Searching, and Purposive Refinement

The review drew on peer‐reviewed empirical studies, reviews, consensus papers, and conceptually informative publications relevant to IBD trial burden, contextual placebo and nocebo effects, specialist IBD nursing, health literacy and therapeutic education, telemonitoring and hybrid follow‐up, transitions in care, fidelity, ethics, and implementation. Literature identification was iterative and purposive rather than exhaustive. The aim was to assemble an information‐rich corpus capable of informing five linked domains: (1) trial burden and retention challenges in IBD; (2) contextual placebo and nocebo processes; (3) literacy‐sensitive education and therapeutic support; (4) continuity, telemonitoring, and hybrid contact models; and (5) transitions into, through, and out of trial participation (Table 1).

**TABLE 1:** Preliminary explanatory framework across the standard care–trial–standard care pathway.

Trial context or vulnerable phase	Supportive nursing function	Likely participant‐level process	Plausible trial‐related implication	Risk if absent or poorly aligned
High burden and uncertainty at screening or enrolment	Empathic, realistic, safety‐bounded communication	Greater trust, clearer expectations, more coherent sense‐making	Better screening persistence, less early disengagement, and reduced misattribution of common sensations	Over‐reassurance, mixed messages, therapeutic misconception, delayed adverse‐event reporting, and lack of compliance
Low health literacy, high protocol complexity, or digital burden	Teach‐back, repetition across touchpoints, micro‐modular education	Stronger understanding, self‐efficacy, and practical task confidence	Fewer misunderstandings, fewer missed assessments, better adherence to protocol tasks	Information overload, one‐off consent teaching, jargon, and silent misunderstanding
Remote monitoring, hybrid visits, fluctuating symptoms	Structured contact plan, symptom triage, defined escalation pathway	Felt continuity, responsive problem‐solving, accountability	Better retention, fewer missed windows, and earlier contact when problems arise	Alarm fatigue, surveillance burden, role confusion, digital exclusion
Trial entry, protocol amendment, treatment switch, or trial exit	Structured onboarding, re‐briefing, end‐of‐study debriefing, written handover, short‐term safety‐net contact	Orientation, closure, reduced abandonment, preserved continuity	Smoother transitions, less avoidable withdrawal, better re‐entry into routine care	Abrupt rupture, confusion, perceived abandonment, disengagement from follow‐up
Multi‐site delivery, diverse participant needs, variable staffing or access barriers	Shared scripts, clear delegation, documentation pathways, equity accommodations, red‐flag escalation rules	Legitimacy, consistency, accessibility, safer support boundaries	More consistent delivery, broader reach, fairer participation, stronger implementation credibility	Scope creep, inequity, inconsistent delivery, safety drift

Searches were conducted between January and March 2026 (updated in April 2026) and primarily covered literature published between January 2010 and March 2026, although earlier landmark publications were retained when considered conceptually essential. The searches generated approximately 240 potentially relevant records, which were progressively refined through purposive screening of which 42 were retained. Sources were not selected according to predefined quality thresholds but through purposive sampling aimed at maximizing conceptual richness, methodological credibility, and explanatory relevance to the review question (Table 2).

**TABLE 2:** Summary and characteristics of the evidence corpus included in the focused narrative review.

Evidence domain	Publications, *n*	References	Predominant evidence designs	Main topics addressed	Contribution to the interpretive synthesis
IBD trial burden, recruitment, retention, and contextual expectation processes	7	[4, 5, 6, 8, 22, 23, 24]	Narrative and systematic reviews, meta‐analysis of randomized trials, patient‐perspective study, and large observational study of screened participants	Recruitment barriers, protocol burden, screen failure, transparency, trust, treatment expectations, symptom attribution, adverse‐event reporting, and discontinuation	Supported the identification of screening and enrolment as vulnerable phases and informed Proposition 1 on empathic, realistic, and safety‐bounded expectation‐shaping. Placebo and nocebo evidence was interpreted as supporting the potential contextual relevance of communication rather than a direct causal effect of nursing support.
Health literacy, education, teach‐back, adherence, and self‐efficacy	6	[25, 26, 27, 28, 29, 30]	Systematic and narrative reviews, educational studies, and randomized intervention studies	Health literacy barriers, therapeutic education, teach‐back, medication adherence, comprehension, and self‐efficacy	Supported Proposition 2 on literacy‐sensitive education and the potential role of teach‐back in identifying misunderstanding before it contributes to protocol deviations, missed assessments, or avoidable withdrawal.
Telemonitoring, remote care, hybrid continuity, and digital equity	10	[31, 32, 33, 34, 35, 36, 37, 38, 39, 40]	Randomized and pragmatic trials, systematic and narrative reviews, qualitative studies, and equity‐focused conceptual literature	Remote monitoring, telemedicine, digital health, continuity of contact, usability, digital burden, access barriers, disparities, triage, and escalation pathways	Supported Proposition 3 on structured hybrid contact. The evidence highlighted the potential value of clear contact pathways and responsive follow‐up while also identifying digital exclusion, surveillance burden, message overload, and unclear escalation processes as implementation risks.
Transitions, nursing roles, governance, safety, and equity	10	[41, 42, 43, 44, 45, 46, 47, 48, 49, 50]	Systematic review, multicenter observational study, mixed‐methods program‐development study, qualitative studies, position statements, consensus guidance, and conceptual papers	Trial entry and exit, pediatric‐to‐adult transition, structured handover, informed consent, professional responsibilities, Clinical Research Nursing, minority recruitment, role clarity, safety, and ethical governance	Informed Propositions 4 and 5 and the STITCH architecture. The evidence supported the interpretation of transitions as vulnerable points requiring structured onboarding, re‐briefing, handover, and safety‐net contact, while identifying governance, role clarity, safety, and equity as enabling conditions for consistent nursing‐led support.
Complex‐intervention development, implementation, and intervention fidelity	9	[51, 52, 53, 54, 55, 56, 57, 58, 59]	Methodological guidance, realist‐methodology papers, scoping reviews, intervention studies, and fidelity‐methodology research	Complex‐intervention development, contextual interpretation, core and adaptable components, delivery fidelity, participant engagement, implementation outcomes, and measurement	Informed the translation of the interpretive propositions into the preliminary PIPN‐IBD and STITCH intervention architectures and supported the specification of candidate components, implementation conditions, and potential fidelity indicators.
Total	42	—	Heterogeneous empirical, review, conceptual, and methodological literature	—	Provided the evidence corpus for the focused interpretive synthesis and the development of five preliminary explanatory propositions.

*Note:* Publications were grouped according to their principal contribution to the review question and interpretive framework. Although some sources addressed more than one topic, each publication was assigned to one predominant evidence domain to avoid double counting.

Publications were considered eligible when they addressed one or more of the following domains: IBD clinical trials, specialist or clinical research nursing, participant engagement, health literacy, placebo or nocebo mechanisms, telemonitoring, continuity of care, transitions, implementation, or intervention fidelity. Editorials without conceptual relevance, conference abstracts lacking sufficient methodological detail, non‐English publications, and studies unrelated to clinical trial participation or nursing support were not included.

Recent literature was prioritized, while older seminal or conceptually important sources were retained where necessary. Adjacent chronic‐care and clinical research literature was included when IBD trial‐specific evidence was limited and when it offered relevant insight into trust, sense‐making, continuity, adherence, transition, or implementation.

### 2.4 Selection and Appraisal

Sources were retained when they offered conceptual, empirical, or practical insight relevant to at least one part of the developing interpretive framework. Rather than applying formal review‐stage appraisal procedures associated with systematic or realist synthesis methodology [51], sources were considered according to three pragmatic questions: whether they were relevant to the review question, whether their claims appeared methodologically credible, and whether they contributed explanatory depth. Particular value was placed on sources that helped clarify under what conditions nursing support was more or less likely to influence participant experience and trial‐related processes.

When evidence was heterogeneous or apparently conflicting, studies were interpreted according to their methodological characteristics, clinical context, and explanatory contribution rather than by attempting quantitative reconciliation. Greater interpretive weight was given to consistent findings across multiple sources while preserving important conceptual differences where appropriate.

### 2.5 Synthesis Output

The final synthesis consisted of realist‐informed explanatory propositions and a preliminary conceptual framework. These findings were also translated into candidate intervention architectures, notably the PIPN‐IBD bundle and the STITCH transition module. These are presented as theory‐informed design propositions rather than pre‐validated solutions [52].

## 3 Results

### 3.1 Overview of the Evidence Domains

The reviewed literature clustered around five overlapping domains: IBD trial burden and retention challenges; contextual placebo and nocebo effects; specialist IBD nursing and research nursing roles; hybrid and decentralized follow‐up models; and transition, fidelity, equity, and governance literature. Across these domains, nursing‐led support did not appear to operate through a single active mechanism. Rather, the literature pointed to relational, educational, cognitive, and organizational functions whose relevance varied according to participant burden, disease volatility, service design, digital demands, and role clarity.

This shifts interpretation away from a simple “nurse present versus nurse absent” comparison. Nursing‐led support appears most relevant when it may help participants interpret uncertainty, understand expectations, remain connected to responsive support, and navigate transitions without abrupt fragmentation. It may contribute to conditions under which trial participation becomes more understandable, manageable, and sustainable.

### 3.2 Propositions

**FIGURE 1:**
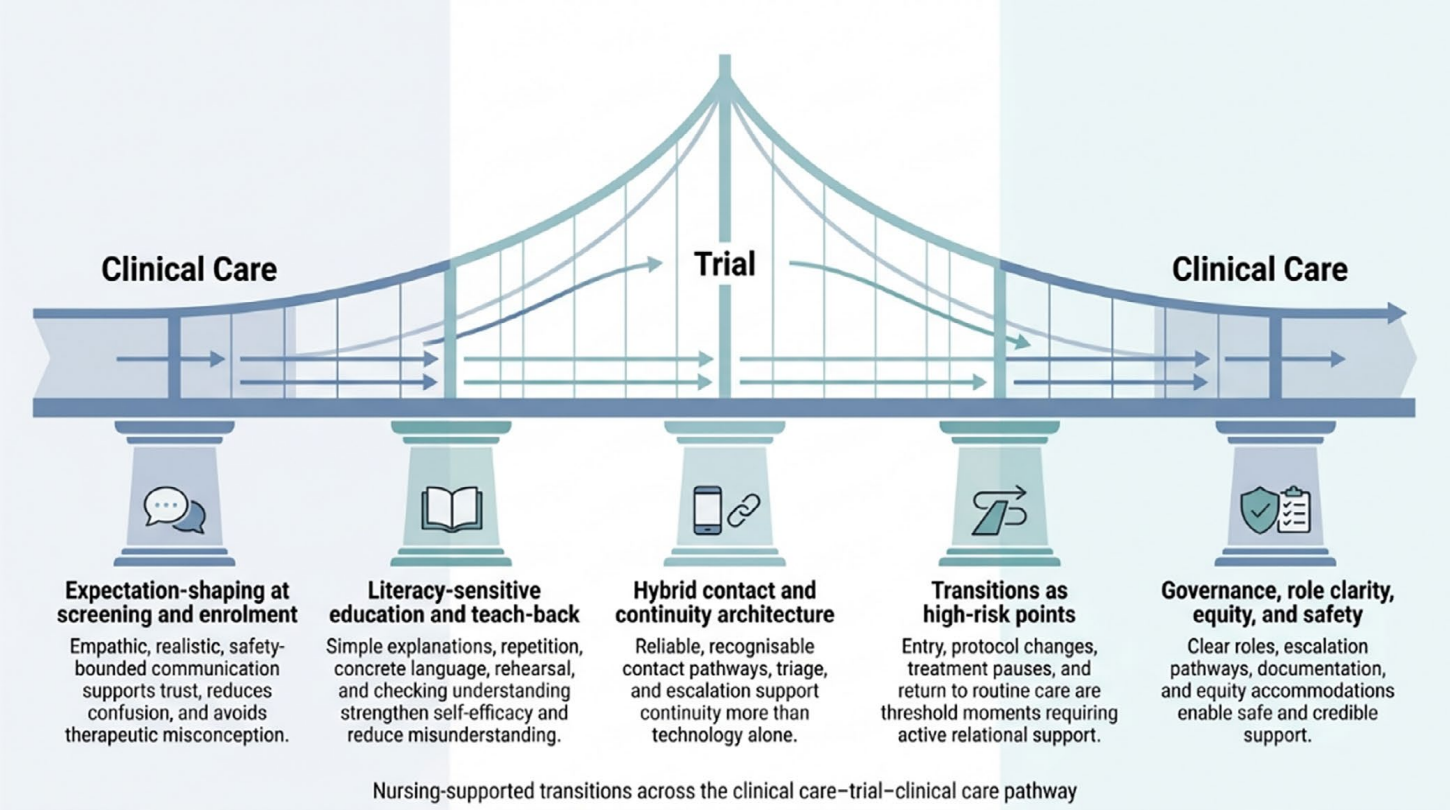
Conceptual model of nursing‐supported transitions across the clinical care–trial–clinical care pathway. The clinical trial is represented as a transitional bridge between routine clinical care before and after participation. Five interrelated supportive mechanisms—expectation‐shaping at screening and enrolment; literacy‐sensitive education and teach‐back; hybrid contact and continuity architecture; recognition of transitions as high‐risk points; and governance, role clarity, equity, and safety—function as structural pillars that sustain continuity, mitigate uncertainty, and support participant engagement. Together, these mechanisms illustrate how nursing contributions operate across thresholds of care to enable coherent, safe, and equitable participation throughout the trial trajectory.

Where possible, supportive functions were interpreted in light of established constructs, including self‐efficacy, sense‐making, therapeutic alliance, and continuity of care. Five preliminary propositions are illustrated in Figure 1.

#### 3.2.1 Preliminary Explanatory Proposition 1. Expectation‐Shaping at Screening and Enrolment

Expectation‐shaping emerged as especially relevant at screening and enrolment, when IBD trials may be experienced as uncertain, burdensome, and risky because of placebo exposure, innovative drugs, washout periods, repeated procedures, and possible screen failure [3, 22, 23, 60]. Nursing communication may be particularly valuable when it is empathic, realistic, and grounded in clear safety boundaries. By reducing confusion and supporting trust, such communication may help participants develop a more coherent framework for interpreting subsequent trial experiences. However, nursing communication should not be regarded as a direct determinant of placebo or nocebo responses. Rather, it may form part of the broader interpersonal and informational context in which participants develop treatment expectations, interpret bodily sensations, and decide whether, when, and how to report symptoms or adverse events.

Expectation‐shaping is not simply reassurance. Overly optimistic or inconsistent communication may encourage therapeutic misconception, delay adverse‐event reporting, or create fragile forms of retention that collapse under uncertainty [5, 6, 8, 41, 42]. Its value lies in calibrated communication: not minimizing burden, but helping participants make sense of it without catastrophizing.

#### 3.2.2 Preliminary Explanatory Proposition 2. Literacy‐Sensitive Education and Teach‐Back

Many challenges of IBD trial participation can be understood as challenges of comprehension and enactment. Participants must understand randomization, expected timelines of benefit, symptom diaries, biospecimen requirements, digital tools, rescue pathways, and distinctions between flare‐related symptoms, common treatment experiences, and possible adverse events. These demands become more difficult when health literacy is limited, symptoms fluctuate, or digital procedures are added to an already complex pathway [25, 26, 27].

Within this context, literacy‐sensitive education and teach‐back are especially relevant. Short explanations, repetition across touchpoints, concrete language, task rehearsal, and explicit checking of understanding can strengthen self‐efficacy and reduce misunderstanding before it becomes a protocol deviation or avoidable withdrawal [28, 29, 30]. Teach‐back may therefore function as both an educational strategy and a potential means of identifying misunderstandings before they result in protocol deviations or avoidable withdrawal.

#### 3.2.3 Preliminary Explanatory Proposition 3. Hybrid Contact and Continuity Architecture

A third pattern concerned hybrid contact, telemonitoring, and continuity. Across IBD nursing and remote follow‐up studies, hybrid models appeared most valuable when they created a recognizable architecture of contact rather than simply multiplying communication channels [31, 32, 33, 34, 35]. Participants appeared more likely to remain engaged when they knew who to contact, what response to expect, and how symptoms or digital alerts would be interpreted.

The main contribution of hybrid follow‐up is therefore not technology itself, but the experience of being held within a responsive and accountable relationship. When contact cadence, triage, and escalation pathways are clear, hybrid follow‐up may contribute to reducing uncertainty, support problem‐solving, and prevent disruptions from becoming missed windows, unplanned service use, or drop‐out. However, digital follow‐up can become burdensome when excessive, poorly bounded, or inequitable. Alarm fatigue, surveillance burden, message overload, low digital confidence, and limited connectivity may weaken continuity. Clear instructions and fewer contact points may speed feedback. Email can offer rapid access and help route questions to the right professional, while phone contact should remain available for urgent matters, including possible serious adverse events [36, 37, 38].

#### 3.2.4 Preliminary Explanatory Proposition 4. Transitions as High‐Risk Points in the Clinical Trial Pathway

The most consistent interpretive pattern concerned transitions. Trial entry, protocol amendments, treatment pauses or switches, and return to routine care function as threshold moments in which participants must renegotiate meaning, expectations, roles, and continuity. These moments were associated with uncertainty, anxiety, perceived loss of control, and risk of disengagement [15, 20, 43, 44, 45]. The informed consent process is therefore crucial: time to read documents, discuss participation with general practitioners, family, or caregivers, and ask questions can empower patients to self‐advocate.

Where transitions were supported through structured onboarding, rebriefing, explanation of changing routines, end‐of‐study debriefing, written handover, and short‐term safety‐net contact, participants may be less likely to experience abandonment or abrupt rupture [19, 21, 46]. They are clinically and relationally significant events in which continuity cannot be assumed and must be actively reconstructed. To ensure an effective transition from trial care to standard care, nurses in routine care may be informed of the transition, with active verbal and written handovers.

#### 3.2.5 Preliminary Explanatory Proposition 5. Governance, Role Clarity, Equity, and Safety as Enabling Conditions

Governance, safety, and equity are not downstream implementation concerns; they enable supportive strategies to operate consistently and safely. Clear delegation, documentation pathways, escalation protocols, shared scripts, and defined scope boundaries stabilize delivery and reduce ambiguity for participants and staff [10, 41, 47, 48, 49].

Equity accommodations work similarly. Language support, alternatives to app‐based workflows, flexible scheduling, disability accommodations, and explicit checks for literacy or digital barriers help ensure supportive mechanisms remain available to participants who might otherwise be excluded [39, 40, 50]. Safety is equally central: without low‐threshold escalation and explicit red‐flag pathways, reassurance or symptom normalization may delay adverse‐event recognition. Governance and equity appear to represent important enabling conditions of intervention credibility, especially where the help‐line is nurse‐led.

### 3.3 Preliminary Explanatory Framework

Together, these propositions support a framework in which nursing‐led support appears most likely to contribute to IBD trial processes when it provides three interrelated resources: (i) an interpretive frame for uncertainty, (ii) practical competence for protocol enactment, and (iii) continuity across vulnerable transitions. Participant engagement may depend not only on the availability of support but also on whether that support helps individuals understand what is happening, what is expected, and how to remain connected when burden and uncertainty are high.

This interpretation reframes engagement, adherence, and retention. Rather than treating them only as operational endpoints, they may be understood as plausible downstream manifestations of trust, self‐efficacy, continuity, and relational security under demanding trial conditions [2, 4, 22].

### 3.4 Implications for Intervention Design

The reviewed literature informed two candidate, theory‐informed intervention architectures. PIPN‐IBD is centered on expectation‐shaping, literacy‐sensitive education, hybrid contact, symptom sense‐making, and standardized escalation support. STITCH focuses on onboarding, amendment rebriefing, end‐of‐study debriefing, structured handover, and time‐limited post‐trial safety‐net contact. Future work should distinguish between core components, essential for mechanism activation, and adaptable elements, in line with complex intervention guidance [53, 54].

Their value lies in making visible what might otherwise remain diffuse. Rather than describing nursing support generically, they specify resources that may plausibly improve continuity, adherence, and perceived abandonment. In this sense, PIPN‐IBD and STITCH are structured candidates for refinement through expert consensus, feasibility testing, and empirical evaluation [53, 54, 61, 62].

### 3.5 Data Integrity, Fidelity, and Implementation Implications

Engagement, adherence, and retention should not be treated only as administrative concerns. When participants trust the trial process, understand procedures, and remain connected to responsive support, they may be more likely to complete assessments, remain within protocol windows, respond to rescue contacts, and report symptoms in a timely way. These behaviors have direct implications for missingness, attrition bias, and outcome interpretability [1, 22, 24].

This also affects fidelity. In this framework, fidelity is the extent to which active ingredients of nursing support, such as expectation‐shaping, teach‐back, and continuity, are delivered in a usable, meaningful form for participants, rather than mere adherence to procedural scripts. Indicators might include teach‐back documentation, completion of onboarding and debriefing, timing of contact around high‐risk transitions, escalation logs, and documentation of contact modality and dose. Implementation outcomes such as reach, feasibility, acceptability, sustainability, and equity remain central because understaffing, digital exclusion, and role confusion may attenuate the mechanisms the intervention is intended to support [55, 56, 57, 58, 59].

## 4 Discussion

This focused narrative review with realist‐informed interpretation suggests that nursing‐led support in IBD trials is best understood as a set of relational, educational, and organizational processes that may help participants navigate uncertainty, maintain continuity, and engage with increasingly complex clinical trial pathways. Nursing may therefore be conceptualized not simply as a delivery role within trials, but as a potentially mechanism‐bearing component of the intervention context that may influence participant reasoning and behavior through contextual and relational processes. Within this framework, nursing communication is not conceptualized as directly generating placebo or nocebo responses. Rather, it may serve as a contextual modifier, influencing how participants understand treatment expectations, interpret bodily sensations, and respond to uncertainty throughout trial participation. Through realistic expectation‐setting, consistent communication, and timely clarification of symptoms, nursing support may help reduce therapeutic misconception, inappropriate symptom attribution, and avoidable nocebo‐related concerns while preserving participant safety and informed decision‐making.

The review also highlights transitions as particularly vulnerable phases of trial participation. Supporting participants during trial entry, protocol amendments, treatment changes, and trial completion may help preserve continuity and reduce avoidable disengagement, suggesting that these moments deserve greater attention in future intervention development [20, 21, 44].

The review also suggests that educational and communicative work may be interpreted more substantively than in many descriptive accounts of nursing roles. Expectation‐shaping, teach‐back, and continuity planning represent core supportive functions rather than optional adjuncts to trial delivery. They may contribute to participants' development of practical competence in remaining engaged with demanding protocols. This is particularly important in IBD, where symptom ambiguity, fluctuating disease activity, digital burden, and placebo/nocebo dynamics complicate participation [3, 5, 25, 63]. Nurses' ability to position patients within a wider network involving clinicians, caregivers, and family may enhance communication, retention, and accountability [64].

Importantly, the present framework focuses on nursing‐led supportive functions rather than on a specific professional title. Depending on local healthcare systems and organizational models, these functions may be delivered by specialist IBD nurses, CRNs, or through collaboration between both roles. Consequently, the proposed framework should be interpreted as describing transferable supportive functions rather than role‐specific responsibilities.

Importantly, the effectiveness of these supportive functions is likely to depend on context. Communication, continuity strategies, and digital support require appropriate governance, clear professional boundaries, and equitable implementation to avoid unintended consequences such as over‐reassurance, surveillance burden, or role ambiguity.

Accordingly, the realist‐informed perspective should be understood as an interpretive approach rather than as the application of formal realist review methodology. It does not provide a completed realist synthesis, formal CMO configurations, or a definitive causal account. Instead, it organizes heterogeneous literature into a preliminary, theory‐informed framework that is clinically intelligible and developmentally useful. The proposed framework is intended to support the design and evaluation of future nursing interventions rather than to provide definitive evidence of causal mechanisms. An important consideration is the transferability of the proposed framework across different healthcare systems. Much of the literature informing this review originates from European settings, where specialist IBD nursing and Clinical Research Nursing roles are relatively well established. In contrast, nursing autonomy, scope of practice, workforce configuration, and governance structures vary substantially across North America, Asia, and lower‐resource healthcare systems. Accordingly, the proposed framework should be interpreted as describing transferable supportive functions rather than a prescriptive organizational model. The specific professionals responsible for delivering these functions and the extent of their autonomy are likely to differ depending on local regulatory frameworks, available resources, and models of care.

Several implications follow. Future nursing interventions in IBD trials should be specified in terms of supportive functions rather than broad role labels. Transition‐sensitive components deserve particular attention in feasibility work [12, 64]. Evaluations should move beyond satisfaction outcomes to include protocol enactment, missed windows, early symptom reporting, continuity indicators, and equity‐sensitive implementation outcomes. Consistent with this, nurse–patient mutuality predicts self‐care behaviors, with specific relational dimensions supporting different domains, suggesting that nursing acts by enhancing patient autonomy rather than merely providing information [65]. Future studies should also report intervention components, implementation context, fidelity, and professional responsibilities with sufficient detail to facilitate replication and comparison across settings. This review has limitations. It was not based on a fully reproducible systematic search strategy and should not be interpreted as exhaustive. Several informative ideas, especially around teach‐back, telemonitoring burden, transition support, and fidelity, were more explicit in adjacent literature than in IBD trial‐specific studies. Evidence on specialist IBD nursing also remains stronger for patient experience and service outcomes than for theory‐informed trial‐process outcomes [12]. Moreover, because most of the included evidence was observational, descriptive, or conceptual, the proposed explanatory propositions should not be interpreted as evidence of causal relationships but rather as plausible mechanisms requiring prospective empirical evaluation. The propositions should therefore be read as hypothesis‐generating and framework‐building rather than definitive.

Overall, the review supports a shift in how nursing‐led support is conceptualized in the IBD clinical trial pathway. Rather than asking only whether nurses improve trial experience, a more useful question is which nursing resources help participants interpret uncertainty, maintain continuity, and complete demanding protocols under real‐world conditions. Framed in this way, the review provides a basis for future consensus work, pilot studies, hybrid evaluations, and intervention testing.

## 5 Conclusion

This review suggests that nursing‐led support in IBD clinical trials should be understood as a contextual and mechanism‐bearing component of participant engagement rather than a solely operational or administrative function. Expectation‐shaping, literacy‐sensitive education, structured continuity, and transition‐focused support may contribute to helping participants interpret uncertainty, maintain protocol adherence, and navigate vulnerable phases of trial participation. The proposed framework highlights the importance of relational continuity, safety, and equity within increasingly complex clinical trial pathways. The specific implementation of these supportive functions is likely to differ depending on the respective responsibilities of specialist IBD nurses and CRNs, local regulatory frameworks, workforce configuration, and resource availability across healthcare systems.

## Funding

The authors have nothing to report.

## Ethics Statement

The authors have nothing to report.

## Consent

The authors have nothing to report.

## Conflicts of Interest

The authors declare no conflicts of interest.

## Supporting information


**Data S1:** jgh370458‐sup‐0001‐Supinfo.docx.

## Data Availability

Data sharing not applicable to this article as no datasets were generated or analysed during the current study.
