# Supplementary material for: Nursing‐Led Support Across the Standard Care–Clinical Trial–Standard Care Pathway in Inflammatory Bowel Disease: A Review With Realist‐Informed Interpretation
Source: JGH Open. 2026 Aug 19;10(8):e70458. doi: 10.1002/jgh3.70458 (PMC13490845; doi:10.1002/jgh3.70458)
Supplement: Supplementary file 1 [file JGH3-10-e70458-s001.docx]

# SANRA reporting/quality checklist for narrative review submission

Manuscript: Nursing-led support across the standard care–clinical trial–standard care pathway in inflammatory bowel disease: a review with realist-informed interpretation

## Checklist mapping

| **Item** | **Text / action** |
| --- | --- |
| 1. Justification of the article's importance for the readership | Addressed in Introduction: IBD trial burden, enrolment/retention challenges, transitions between care and research, and nursing contribution. |
| 2. Statement of concrete aims or formulation of questions | Addressed in Introduction and Methods/Review question. |
| 3. Description of the literature search | Addressed in Methods/Evidence corpus, searching, and purposive refinement. Consider adding more exact search strings if the journal requests greater reproducibility. |
| 4. Referencing | Addressed through numbered references. Before submission, verify all DOIs, journal abbreviations, and reference formatting against Wiley style. |
| 5. Scientific reasoning | Addressed through realist-informed propositions, explanatory framework, intervention architectures, and limitations. |
| 6. Appropriate presentation of data | Addressed through Figure 1 and Table 1. |

## Statement for manuscript or submission system

This focused narrative review was prepared with reference to the Scale for the Assessment of Narrative Review Articles (SANRA). Because the review used realist-informed interpretation but did not aim to conduct a formal realist synthesis, RAMESES publication standards were considered as a methodological sensitising reference rather than claimed as a formal reporting standard.
